# The measurement of true initial rates is not always absolutely necessary to estimate enzyme kinetic parameters

**DOI:** 10.1038/s41598-023-41805-y

**Published:** 2023-09-12

**Authors:** Jean-Marie Frère, Olivier Verlaine, André Matagne

**Affiliations:** 1 Enzymology and Protein Folding Laboratory, University of Liège, Building B6C, Quartier Agora, Allée du 6 Août, 13, 4000 Liège (Sart-Tilman), Belgium; 2 Centre for Protein Engineering, InBioS, University of Liège, Building B6C, Quartier Agora, Allée du 6 Août, 13, 4000 Liège (Sart-Tilman), Belgium

**Keywords:** Biochemistry, Biophysics

## Abstract

In the chapters dealing with enzyme reactions, the authors of all Biochemistry textbooks and of even more specialized texts consider that the characteristic parameters (*k*_*cat*_ and *K*_*m*_) must be determined under initial or steady-state rate conditions. This implies the transformation of a very limited proportion of substrate (at most 10–20%) or a continuous recording of the product or substrate concentration vs. time. Both options can present practical difficulties. Is it possible to get around these very stringent conditions? Here we show that in the most favourable cases up  to 70% of the substrate can be converted resulting in systematic errors on the parameters (that can easily be taken account of) if the simple Henri-Michaelis–Menten equation is utilised. Alternatively, the integrated form of the same equation directly yields excellent estimates of the same parameters. Our observations should greatly facilitate the task of researchers who study systems in which measurements of the reaction progress are painstaking or when substrate concentrations close to the detection limit must be used. The general conclusion is that it is not always absolutely necessary to determine initial or steady-state rates to obtain reliable estimations of the enzyme kinetic parameters..

## Introduction

In many enzymatic systems, the relationship between the reaction rate and the substrate concentration is described by the hyperbolic Henri–Michaelis–Menten (HMM) Eq. (1) that allows the determination of the *k*_cat_ and *K*_m_ parameters.

1 $$v=\frac{V\cdot {[\mathrm{S}]}_{0}}{{K}_{m}+{[\mathrm{S}]}_{0}}$$

where *V* = *k*_cat_·[E]_0_, $v$ is the initial or steady-state rate, [S]_0_ the initial substrate concentration and [E]_0_ the total enzyme concentration (that is supposed to be < < [S]_0_). *K*_m_, termed the Michaelis constant, is the concentration of substrate at which *v* = *V*/2, and *V* is the maximum, or more accurately, the limiting rate. In simple systems (single substrate, quasi-irreversible reactions, no inhibition by P or excess S), this equation remains valid along the reaction time course if one replaces *v* by d[P]/dt and [S]_0_ by [S]_0_ – [P] so that Eq. (2) is the integrated form^1^ of Eq. (1):

2 $$t=\frac{\left[\mathrm{P}\right]}{V}+\frac{{K}_{m}}{V}\cdot \mathrm{ln}\left(\frac{{[\mathrm{S}]}_{0}}{{[\mathrm{S}]}_{0}-\left[\mathrm{P}\right]}\right)$$

where [P] is the product concentration at time *t.* The integrated equation sometimes allows to determine *V* and *K*_m_ by analysing complete time-courses^2^.

The definition of the initial or steady-state rate implies that the substrate concentration does not significantly decrease over the reaction time, resulting in stable concentration(s) of the intermediate(s). Thus, $v$ is best defined as ${\left(\frac{\mathrm{d}\left[\mathrm{P}\right]}{\mathrm{dt}}\right)}_{\mathrm{t}=0}$ or ${-\left(\frac{\mathrm{d}\left[\mathrm{S}\right]}{\mathrm{dt}}\right)}_{\mathrm{t}=0}$, where $\left[\mathrm{S}\right]$ and $\left[\mathrm{P}\right]$ are the substrate and product concentrations, respectively.

Real quasi-linear [S] or [P] vs. t curves can only be obtained when [S]_0_ >> *K*_m_, so that, under conditions suitable for the determination of *K*_m_ (i.e. 0.25 *K*_m _≤ [S]_0_ ≤ 4 *K*_m_), the rate decreases as soon as the reaction is started. Extrapolation methods^3,4^ have been described to determine *v* on the basis of the early part of the reaction time-course, for instance by extrapolating [P]/t = f([P]) to [P] = 0, but this requires that the reaction be monitored continuously by e.g. spectrophotometric measurements. Similarly, the “chord method” proposed by Waley^5^ is also based on the evaluation of [P] or [S] at a minimum of two or three *t* values. In many cases, however, the appearance of product or disappearance of substrate can only be followed discontinuously by, for instance, HPLC or other chromatographic or electrophoretic methods, and hence accumulating a sufficient number of time points can become extremely time-consuming and require up-to-date equipment that is not always available.

Usually general Biochemistry textbooks^6–10^ just state that utilization of the HMM equation requires the measurement of the reaction rate during the stationary state phase, or define *v* as the slope of the [P] vs. *t* curve at *t* = 0. More surprisingly, in specialized enzymology books^11–24^, the initial or steady state rate is generally well defined but the way to determine its value in practice remains somewhat enigmatic. Most often, *v* is defined as the rate during the formation of the first percentages of product^13^ (but, depending on the author, this can vary from 1–2%^14^ to 5%^15^, 10%^16^ or even 20%^11^), the tangent to the curve at *t* = 0^17,18^ or the rate as long as the accumulation of product remains linear vs. *t*^20,21^. Hayashi and Sakamoto^22^ provide a detailed and complete analysis of steady-state and rapid equilibrium systems, but do not describe any simple method to determine *v* experimentally. By contrast, Cornish-Bowden^23^ proposes practical methods to determine *v*, for instance Boeker's^3^ [P]/*t* = f([P]) regression, whereas Wong^4^ suggests a more complex approach based on a fit to a polynomial. Finally, a discussion on the validity of the HMM equation can be found^25^, where the “steady-state” and “reactant stationary” assumptions are discussed in the frame of a detailed historical view. Again, the practical aspects of a reliable measurement of *v* are barely mentioned. The best and clearest approach to the evaluation of *V* and *K*_m_ is probably that of Cornish-Bowden, who presents an in-depth analysis of all the pertinent (and non-pertinent) ways to do so.

With the possible exception of the chord method^24^, no mention is ever made of the difficulty of collecting many time-points in cases where [P] or [S] must be evaluated by discontinuous, time-consuming techniques. Rather surprisingly, to our knowledge, the following question does not appear to have been asked: what happens if a relatively large proportion of the substrate is transformed and the [P] or [S] value is determined at one single time-point only? Thus, using the integrated Eq. (2), we performed simulations that enabled an analysis of the behaviours of the *v* and [P]/*t* values as a function of [S]_0_, and provided an evaluation of the systematic errors that are to be expected when increasing values of the [P]/[S]_0_ ratio are used.

The analysis presented here is valid if the following conditions are fulfilled:

1. For sufficiently long incubation times, the reaction is complete or nearly complete for the considered substrate. Note that a reversible reaction can be made irreversible by removing one of the products (if this is impossible, the measurement of initial rates remains the best solution although it might become difficult if the equilibrium constant is significantly lower than 1). In the case of a multi-substrate system, the concentration(s) of the other substrate(s) is (are) such that it (they) can be considered as constant.
2. The enzyme does not lose activity during the incubation time. This can be easily verified with the help of Selwyn's test^26^.
3. There is no inhibition by the product or excess substrate (these situations that can usually be easily detected will be analysed in a further contribution where we plan to present the results of simulations based on the corresponding integrated equations^2^).
4. There is no non-enzymatic disappearance of S.

Clearly, our analysis does not apply in the cases of more complex kinetics. For example positive cooperativity implies equations that are quite different from Eq. (1) (but it can be shown that this phenomenon can also be identified when a large proportion of S is converted). By contrast, detection of substrate-induced activation or inactivation (burst or lag, hysteresis phenomena^27^) requires the monitoring of a time-course, a control that is necessary in all cases. Moreover, in these systems, the “initial” and steady-state rates are not the same. As an example, substrate-induced inactivation occurs very often with class D β-lactamases^28^, and upon the hydrolysis of some rather poor substrates with class C^29^ and class A^30^ β-lactamases.

## Results

### Theory and simulations

In a first approach, we simulated the time-courses of the enzymatic reactions with the help of Eq. (2).

Arbitrary units were used and, to simplify the calculations, the *V* and *K*_m_ values were both set to 1. The substrate concentrations were 0.35, 0.7, 1.05, 1.4, 1.75, 2.1 and 2.45 (all >> [E]_0_). In the first series of simulations, we increased the proportion of substrate conversion from 10 to 50% at all initial substrate concentrations. Table S1 shows examples of these simulations.

On the basis of these data, three reaction rates were calculated. The first one corresponded to [P]/*t* (the chord), the second and third ones to the linear regressions [P] = v_1_·*t* and [P] = *a* + v_2_·*t*, respectively. Not surprisingly, *v*_2_ was in all cases found to be nearly identical to [P]/*t*^4^. These data were then analysed using the HMM Eq. (1), considering that [P]/*t* was an adequate approximation of the steady-state rate, although it was clear that this was not a valid hypothesis. Figure S1 shows the [P]/*t* vs. [S]_0_ plot at 50% transformation of the substrate and Table S2 summarizes the *V*_app_ and (*K*_m_)_app_ values deduced from similar plots for all percentages of substrate conversion. Figure S1 shows that the [P]/*t* vs. [S]_0_ plot shows no significant deviation from a hyperbolic relationship and Table S2 indicates that this is even more true at lower percentages of substrate conversion. The deduced values of *V* (i.e. *V*_app_) are surprisingly correct, while those of *K*_m_ (i.e. (*K*_m_)_app_) are larger than the «real» values (although at 30% of transformation, the overestimation of *K*_m_ remains < 20%) and this overestimation increases with the percentage of substrate conversion. In all cases, the agreement with a hyperbolic relationship remains very good, as indicated by low SE values.

In practice, however, it will probably be quite difficult and time-consuming to adjust the reaction times in order to obtain similar percentages of substrate conversion for all [S]_0_ values. In consequence, we repeated the simulations with identical final *t* values (i.e. identical reaction times) for all substrate concentrations (so that the percentage of substrate conversion expectedly decreased with increasing [S]_0_ values). In this “constant time” strategy, the final *t* values were chosen so that they resulted in 10, 20, 30, 40, 50, 60 and even 70% of substrate transformation at the lowest [S]_0_ concentration. Table S1D shows the simulation at [S]_0_ = 2.45, with a reaction time identical to that necessary to achieve 50% transformation of the substrate at the lowest concentration (i.e. *t* = 0.8681, Table S1B and D). The results in Table 1 were obtained on this basis (at all percentages of substrate conversion), by choosing the [P] values so that the *t* values were the same for all [S]_0_ values. The results of the simulations were analysed both by direct regression using the hyperbolic Eq. (1) and its Hanes–Woolf (HW) linearization (Eq. (3)) (both with [P]/t = *v*, *V*_*app*_ = *V* and (*K*_*m*_)_*app*_ = *K*_*m*_):

**Table 1:** Constant time strategy.

t	% Substrate conversion at [S]_0_ =		*V*_*app*_	*(K*_m_)_app_	*V*_*app/*_*(K*_m_)_app_
	0.35	2.45			
0.1404	10	4.03	1.017 ± 0.004	1.074 ± 0.007	0.947 ± 0.005
0.2941	20	8.4	1.031 ± 0.004	1.148 ± 0.011	0.898 ± 0.005
0.4612	30	13.1	1.057 ± 0.008	1.258 ± 0.019	0.840 ± 0.008
0.6505	40	18.3	1.081 ± 0.010	1.377 ± 0.0309	0.785 ± 0.009
0.8681	50	24.1	1,124 ± 0.014	1.562 ± 0.048	0.719 ± 0.009
1.126	60	30.9	1.182 ± 0.024	1.822 ± 0.072	0.649 ± 0.011
1.450	70	39.0	1.268 ± 0.033	2.210 ± 0.11	0.575 ± 0.011
Hyp	70	39.0	1.233 ± 0.031	2.097 ± 0.094	0.588 ± 0.090

Values of *V*_*app*_ and (*K*_m_)_*app*_ deduced from the HW plots for increasing percentages of substrate conversion. The percentage given in the second column is that simulated at the lowest [S]_0_ value (0.35). For the other [S]_0_ values, [P] values were adjusted so that the final *t* value was the same as that obtained at [S]_0_ = 0.35 (see Table S1 for the simulations with 50% substrate transformation at [S]_0_ = 0.35 (B) and at 2.45 (D) with the same final *t* value, i.e. 0.8681 and for other [S]_0_ values). All units are arbitrary (see text). Direct fitting of the hyperbolic equation to [P]/*t* vs. [S]_0_ values yielded essentially the same results with minor differences (identical values up to 40% and < 5% differences at 50 to 70% substrate conversion: the last line shows the values obtained with the regression according to the hyperbolic equation at 70% conversion). When real *v* values were similarly analysed, the SEs on *V* and *K*_m_ (both equal to 1.00) were respectively 0.1 and 0.3%. This is due to the rounding of the *v* values to 3 significant digits in contrast to the larger SEs observed when increasing percentages of S are converted that reflect increasing deviations from the hyperbola.

3 $$\frac{{[\mathrm{S}]}_{0}}{v}=\frac{{K}_{m}+{[\mathrm{S}]}_{0}}{\mathrm{V}}$$

**Figure 1:**
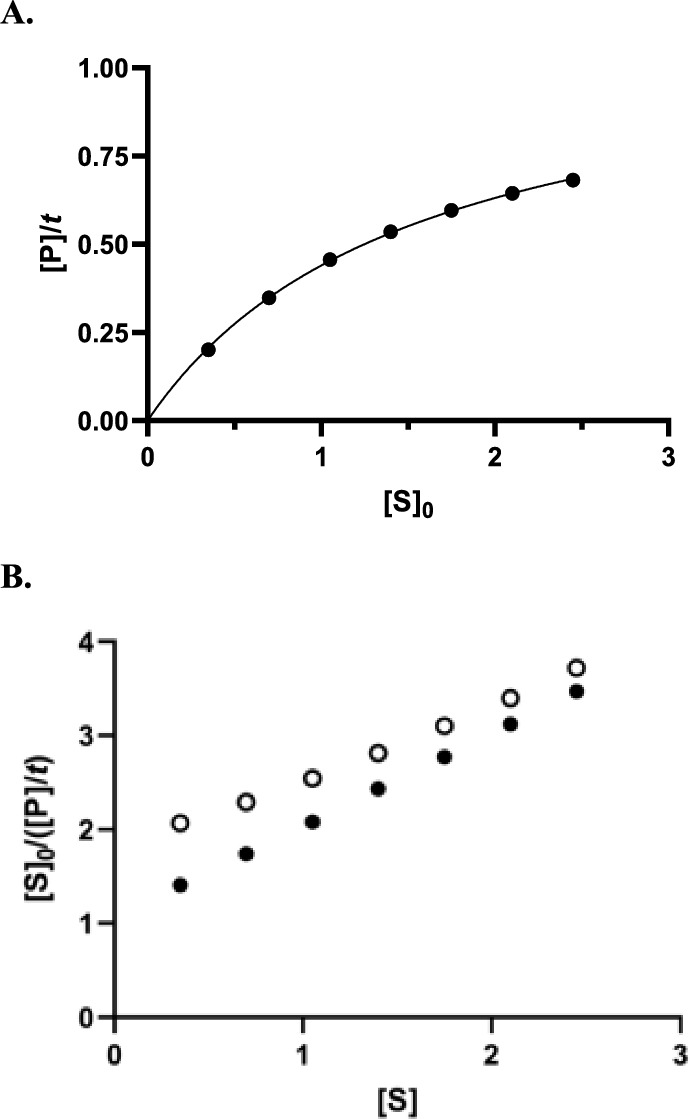
Results obtained by (wrongly) assuming that [P]/t corresponds to the initial rate. Constant time strategy. (**A**) Plot of [P]/*t* vs. [S]_0_ at a *t* value corresponding to that necessary to reach 50% of substrate conversion at the lowest [S]_0_ value. Data were analysed on the basis of the HMM Eq. (1) and the solid line was drawn using the values of the kinetic parameters shown in Table 1. All units are arbitrary (see text). (**B**) Hanes–Woolf (Eq. 3) plots built with the [P]/t values obtained with 70 (○) or 10 (●) % of substrate conversion (at the lowest [S]_0_ value). A slight upward curvature can be detected at 70% conversion, not at 10%.

Both (*K*_m_)_app_ and *V*_app_ are larger in Table 1 than in Table S2 (the former significantly more so than the latter) and much larger SE values are obtained, which indicates larger deviations from a true hyperbolic relationship between [P]/*t* and [S]_0_. However, as shown by Fig. 1, even with 70% substrate transformation at [S]_0_ = 0.35, this deviation is not spectacular. However although the deduced value of *V* remains reasonably correct (1.27 ± 0.033), that of *K*_m_ (2.21 ± 0.11) is more than twice the real one and the SE becomes larger. Although the deviation from a true hyperbola remains difficult to visualize in the [P]/*t* vs. [S]_0_ graph (not shown), the HW plot (Fig. 1B) exhibits a distinct upward curvature. In practice, however, this might remain insignificant in the presence of experimental errors (see Fig. S2). Fitting the HMM hyperbolic equation or its HW linearisation always yielded essentially the same results so that, in the following text, the latter was preferred when applying the simple HMM model in order to more easily detect possible deviations that always resulted in upward curvatures in the linear plots.

Note that, in all cases, the v_2_ values gave data that were almost indistinguishable from those obtained with [P]/*t*, while v_1_ values resulted in somewhat better fits. However, since our goal was to use a very small number of time points (just one if possible), this option was not realistic for the purposes of the present analysis. In consequence, if [P] is measured at only one time point, the only relevant experimental value is [P]/*t*. The *V*_*app*_ and *(K*_*m*_*)*_*app*_ values obtained by (erroneously) assuming that the [P]/*t* values represent the «initial» rates can easily be corrected on the basis of Table 1 yielding the correct values of *V* and* K*_m_.

Finally, in order to see if it was possible to obtain better estimates of the kinetic parameters on the basis of the same data, we used Eq. (2) rearranged as follows:

4 $$\frac{\left[\mathrm{P}\right]}{t}=V-\frac{{K}_{m}}{t}\cdot {\text{ln}}\left(\frac{[{\mathrm{S}]}_{0}}{{[\mathrm{S}]}_{0}-\left[\mathrm{P}\right]}\right)$$

so that *V* and *K*_m_ can easily be obtained by plotting $\frac{\left[\mathrm{P}\right]}{\mathrm{t}}$ vs. $\frac{1}{\mathrm{t}}\cdot {\text{ln}}\left(\frac{{[\mathrm{S}]}_{0}}{[{\mathrm{S}]}_{0}-\left[\mathrm{P}\right]}\right)$.

Not surprisingly, when Eq. (4) or the equivalent Eq. (2) is used to analyse the simulated data, the starting values are recovered for both *V* and *K*_m_ (i.e. $1$.0 and $1.0$). In practice, it seems useful to compare the values obtained with the rigorous Eq. (4) to those determined from the fitting of [P]/*t* vs. ${[\mathrm{S}]}_{0}$ according to the hyperbolic HMM Eq. (1) (see “Discussion”).

We also performed simulations using substrate concentration ranges below (i.e. 0.07–0.35, by steps of 0.07) or above (i.e. 1.4–8.4, by steps of 1.4) the *K*_m_ value. As expected, with the values obtained at 50% transformation of the substrate at the lowest [S]_0_ (i.e. constant time strategy), when all [S]_0_ values were < *K*_m_, the overestimations and the deviations from a true hyperbola became larger and we calculated *V*_app_ = 1.30 ± 0.09 and (*K*_m_)_app_ = 1.82 ± 0.12. By contrast (and somewhat unexpectedly, at least for us), when all the [S]_0_ values were above *K*_m_, the overestimations (*V*_app_ = 1.05 ± 0.03 and (*K*_m_)_app_ = 1.46 ± 0.17) were even smaller than in the case where [S]_0_ ranged from 0.35 to 2.45 (see Tables 2, 3), but the deviation from the hyperbola, as characterised by the SE value on *K*_m_, was more pronounced.

**Table 2:** Summary of the results of simulations with errors (Hanes–Woolf plots).

% of S conversion	Range of values			Largest SE (%)			Averages of 18 runs		
	*V*_app_	(*K*_m_)_app_	*V*_app_/(*K*_*m*_)_*app*_	*V*_app_	(*K*_m_)_app_	*V*_app_/(*K*_m_)_app_	*V*_app_	(*K*_m_)_app_	*V*_app_/(*K*_m_)_app_
Relative random errors up to 5% (18 runs)									
Real v	0.92–1.12	0.83–1.26	0.89–1.11	6	14	9	1.02 ± 0.04	1.03 ± 0.10	0.99 ± 0.04
10	0.95–1.09	0.92–1.25	0.87–1.03	6	14	9	1.02 ± 0.04	1.10 ± 0.09	0.94 ± 0.06
30	0.99–1.12	1.11–1.41	0.79–0.89	6	17	9	1.06 ± 0.05	1.26 ± 0.13	0.84 ± 0.03
60	1.03–1.38	1.18–2.10	0.62–0.71	6	11	5	1.17 ± 0.05	1.77 ± 0.16	0.66 ± 0.04
Relative random errors up to 10% (18 runs)									
Real v	0.86–1.27	0.76–1.57	0.81–1.15	11	28	20	1.03 ± 0.08	1.08 ± 0.21	0.97 ± 0.07
10	0.85–1.20	0.68–1.51	0.80–1.25	12	29	19	1.02 ± 0.08	1.08 ± 0.21	0.96 ± 0.07
30	0.97–1.20	1.00–1.59	0.75–0.98	11	27	14	1.05 ± 0.08	1.25 ± 0.21	0.84 ± 0.05
60	0.96–1.69	1.19–3.14	0.54–0.81	14	25	11	1.25 ± 0.12	1.98 ± 0.33	0.64 ± 0.05
Absolute random errors up to 20% of the value recorded at the lowest [S]_0_ (18 runs)									
Real v	0.88–1.12	0.68–1.40	0.76–1.33	11	25	15	1.02 ± 0.08	1.04 ± 0.20	1.00 ± 0.12
10	0.91–1.13	0.79–1.41	0.82–1.18	12	28	18	1.03 ± 0.08	1.10 ± 0.20	0.95 ± 0.11
30	0.93–1.22	0.89–1.70	0.71–1.05	11	22	12	1.03 ± 0.08	1.21 ± 0.20	0.87 ± 0.08
60	1.08–1.41	1.42–2.50	0.56–0.72	16	25	11	1.22 ± 0.11	1.91 ± 0.33	0.65 ± 0.05

**Table 3:** Summary of the results of simulations with errors (integrated Eq. 2).

% of S conversion	Range of values		Largest SE (%)		Averages of 18 runs	
	*V*_app_	(*K*_m_)_app_	*V*_app_	(*K*_m_)_app_	*V*_app_	(*K*_m_)_app_
Random errors up to 5% (18 runs)						
Real v*	0.93–1.06	0.85–1.23	5	9	1.01 ± 0.04	1.01 ± 0.07
10	0.93–1.06	0.88–1.15	5	11	1.00 ± 0.04	1.00 ± 0.07
30	0.94–1.02	0.91–1.13	6	12	1.00 ± 0.04	0.99 ± 0.07
60	0.91–1.11	0.85–1.11	4	7	1.00 ± 0.03	0.97 ± 0.05
Random errors up to 10% (18 runs)						
Real v*	0.86–1.21	0.71–1.41	10	20	1.01 ± 0.08	1.02 ± 0.16
10	0.86–1.14	0.67–1.33	12	26	0.99 ± 0.07	0.99 ± 0.14
30	0.90–1.08	0.77–1.14	9	19	0.98 ± 0.06	0.95 ± 0.12
60	0.86–1.27	0.74–1.52	10	19	1.01 ± 0.07	1.01 ± 0.13
Absolute random errors up to 20% of the value recorded at the lowest [S]_0_ (18 runs)						
Real v*	0.88–1.13	0.69–1.33	14	28	1.00 ± 0.08	0.99 ± 0.17
10	0.87–1.13	0.95–1.31	14	30	1.00 ± 0.08	0.99 ± 0.17
30	0.88–1.13	0.68–1.32	13	25	0.95 ± 0.08	0.90 ± 0.15
60	0.81–1.16	0.60–1.39	18	32	0.97 ± 0.09	0.93 ± 0.17

*Values computed based on a very short time (0.001). In the absence of errors (rounding errors < 0.5%), the expected V and Km values were retrieved with SE’s < 1%.

In some cases, it can be interesting to directly measure the *V*/*K*_m_ value at [S]_0_ < *K*_m_. On the basis of Eqs. (2) and (4), we computed the (*V*/*K*_m_)_app_ values at [S]_0_ = 0.35 assuming that [P]/*t* represented the initial rate and that [S]_0_ was < < *K*_m_ (both assumptions being clearly wrong). With 10, 30 and 60% substrate conversion, the (*V*/*K*_m_)_app_ values were respectively 71, 65 and 53% of the real *V*/*K*_m_ value. At [S]_0_ = 0.1 *Km*, these values were respectively 87, 78 and 61%. Again, these underestimated values can be corrected with the help of Table 1. It is thus possible to obtain reasonable approximations of *V*/*K*_m_ with one single point at [S]_0_ < 0.4 *Km* and with a rather large degree of substrate conversion.

### Introduction of experimental errors

In order to better mimic experimental conditions, we introduced random errors of up to 5 and 10% on the simulated values or absolute errors corresponding to 20% of the value obtained at the lowest [S]_0_. To obtain a valid comparison, we similarly introduced errors on the true initial rates computed according to the HMM equation. The results were analysed based on the hyperbolic equation, the HW plot and the integrated equation, and are summarized in Tables 2 and 3. 18 simulations were performed for each percentage of substrate transformation and each error level. To show that 18 simulations were providing a representative sample of the vast number of possibilities, we performed 5 separate sets of 18 simulations that yielded very similar results (Table S3).

When these results are analysed with the help of the HW plot (or on the basis of the hyperbolic equation, see legend to Table 1) with the (wrong) assumption that [P]/*t* = *v*, the following observations can be made:

- With all the % of substrate conversion, there are no major differences between the average (*V)*_*app*_ and (*K*_m_)_app_ values determined with [P]/*t* and those obtained with no error shown in Table 1.
- Up to 30% of substrate conversion, there is no major difference in the ranges of the (*V)*_*app*_ and (*K*_m_)_app_ values when determined based on the real *v* or the [P]/*t* values. The overestimation of *V* (*V*_app_) remains negligible and that of *K*_m_ ((*K*_m_)_app_) reasonable (≤ 40%). The SEs are not significantly higher than with real *v’*s (Tables 1 and 2).
- At 60% conversion, the range is a little larger, but not dramatically so. For instance, with errors up to 10% and for the *K*_m_ values (that exhibit a larger degree of variation than *V*), the integrated equation yields a range of 0.74–1.52 with 60% conversion vs. 0.71–1.41 with *v* values. With the HW linearization, account must be taken of the overevaluation depicted in Table 1 so that the ranges are 0.65–1.73 at 60% conversion vs. 0.76–1.57 with *v* (compare Tables 1,  2 and 3).
- When the integrated equation is used, all the results give values in good agreement with the theoretical ones and the errors are not larger with 60% conversion of the substrate than with the real *v* values.

In conclusion, good values of *V* and *K*_m_ can be derived from experiments in which large percentages of the substate are transformed. If [P]/*t* values are utilised for building a HW plot, the overestimation of *V* remains reasonable while that of *K*_m_ is somewhat larger, but these systematic errors can easily be corrected with the help of the integrated equation (or by correcting on the basis of Table 1). The experimental error (5 or 10%) not unexpectedly represents the most important factor in determining the quality of the results and this is clearly also true when the real *v* values are measured.

### Experimental studies

**Figure 2:**
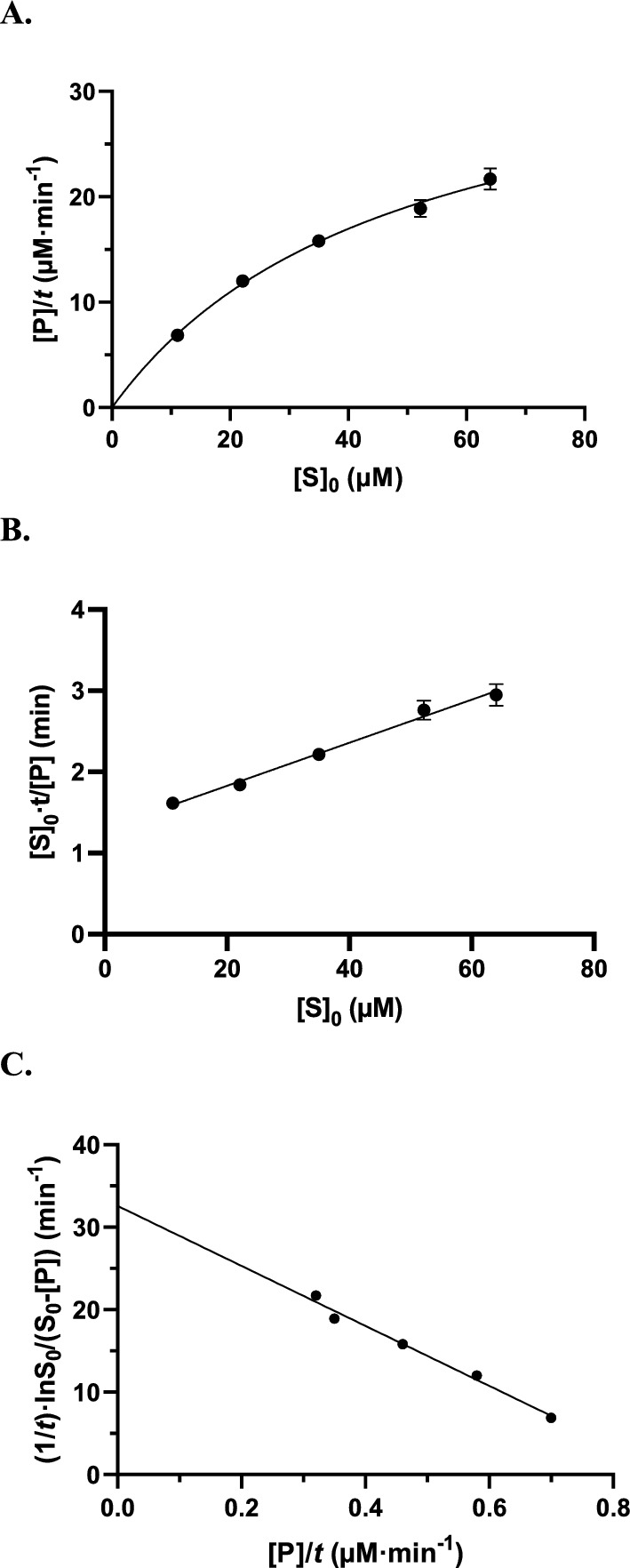
Hydrolysis of nitrocefin by the P99 β-lactamase. (**A**) Plot of [P]/*t* as a function of [S]_0_ with t = 48.9 s. 51.2% of the substrate were hydrolysed at the lowest concentration (i.e. 11.1 µM, see Table S4). (**B**) Hanes–Woolf plot of the same data. (**C**) Integrated equation. Plot of [P]/t vs. $\frac{1}{t}\cdot {\text{ln}}\left(\frac{{[\mathrm{S}]}_{0}}{{[\mathrm{S}]}_{0}-\left[\mathrm{P}\right]}\right)$ (Eq. (4)) with the same data.

Reaction time-courses were determined with the *Enterobacter cloacae* P99 class C β-lactamase and nitrocefin at concentrations in the range of 11 to 64 µM. The published^31^ *k*_cat_ and *K*_m_ values are 780 s^−1^ and 25 µM, respectively. The reaction was followed for 66 to 75 s and, since the mixing dead-time (5–10 s) was not negligible when compared to the total duration of the reaction monitoring, the exact initial substrate concentration ([S]_0_) at the time of the first measurement (i.e. *t* = 0) was computed by subtracting the reading at the first time-point from the final one. The time point corresponding to about 50% substrate conversion at the lowest concentration was determined (48.9 s) and the concentrations of product obtained after the same time were recorded at the other substrate concentrations. It is important to note that, although many data points (2 readings/s) were accumulated, only one was used in the calculations (for calculation of [P]/*t*, see above). Table S4 summarises the results of a total of 18 experiments at 5 different initial substrate concentrations. Then, [P]/*t* was plotted vs. [S]_0_ yielding the graph presented in Fig. 2A and the hyperbolic fitting yielded *V*_app_ = 37 ± 1.3 µM/min and (*K*_m_)_app_ = 49 ± 2.7 µM. The HW linearization (Fig. 2B) yielded essentially the same results.

Equation (4) was then used to analyse the same data (Fig. 2C), yielding the «correct» values for the kinetic parameters, namely *V* = 33 ± 1.3 µM/min and *K*_m_ = 29 ± 4 µM. Since we had recorded time-courses up to at least 1 min, we repeated the calculations at 25 and 60% of substrate transformation at the lowest [S]_0_ value. The results are summarized in Table 4. As expected on the basis of the simulations (see Table 1), the *V*_app_ and (*K*_m_)_app_ values increased with the percentage of substrate conversion. The *V* and *K*_m_ values calculated with the help of Eq. (4) were remarkably consistent and the *V*_app_/*V* and (*K*_m_)_app_/*K*_m_ ratios nicely reflected those deduced from the simulations. The average values were *V* = 33.2 ± 1.4 µM/min (*k*_cat_ = 920 ± 40 s^−1^ ) and *K*_m_ = 31 ± 3 µM, in good agreement with those determined before (*k*_cat_ = 780 ± 30 s ^1^ and *K*_m_ = 25 ± 1 µM)^31^ under slightly different conditions.

**Table 4:** **S**ummary of experimental results for the hydrolysis of nitrocefin by the P99 β-lactamase and comparison with the simulations.

% Substrate conversion^a^	On the basis of Eq. (1) or (3)		On the basis of Eq. (4)		Ratios Eq. (1)/Eq. (4)	
	(*K*_m_)_app_ (µM)	*V*_app_ (µM min^−1^)	*K*_m_ (µM)	*V* (µM min^−1^)	(*K*_m_)_app_/*K*_m_	*V*_app_/*V*
25	39.4 ± 5.0	36.2 ± 2.2	32.4 ± 4.0	34.3 ± 2.3	1.22 ± 0.15 (exp: 1.20)^b^	1.06 ± 0.07 (exp: 1.05)^b^
50	49 ± 2.7	37 ± 1.3	29 ± 4	33 ± 1.3	1.66 ± 0.10 (exp: 1.54)^b^	1.12 ± 0.04 (exp: 1.12)^b^
60	55.5 ± 5.8	39.2 ± 2.3	28.5 ± 3.0	31.5 ± 2.0	1.95 ± 0.22 (exp: 1.81)^b^	1.24 ± 0.07 (exp: 1.18)^b^

Since the reaction time-courses had been continuously monitored, quasi-initial rates could also be determined (at 5 and 10% substrate conversion). Analysis of these data with the HW linearisation yielded the following results: at 5% conversion, *V* = 35 ± 2.3 µM min^−1^ and *K*_*m*_ = 35 ± 7 µM; at 10% conversion, *V* = 34 ± 2.2 µM min^−1^ and *K*_*m*_ = 35 ± 5 µM.

^a^At the lowest [S]_0_ value.

^b^Expected on the basis of Table 1.

## Discussion

Our simulations indicate that good estimates of *V* and *K*_m_ can be obtained with the HMM equation by (erroneously) considering that *v* = [P]/*t* up to 50% of substrate transformation at the lowest [S]_0_ value (Tables 2, 3). Even at 70%, the *K*_m_ and *V* values are overestimated only 2.2- and 1.25-fold, respectively. In all cases, the value of *V* is much closer to the real one than that of *K*_m_. Furthermore, our analysis shows that the estimation of the kinetic parameters can be significantly improved with the help of the integrated Eq. (4) and it is important to underline the fact that this can be done by utilising the same experimental results. It is also noteworthy that the results obtained at 10% substrate conversion or with real initial rate (*v*) values were nearly identical when the HW linearisation was used.

Interestingly, Lee and Wilson^32^ suggested an approach where (up to 50% substrate conversion) they replaced [S]_0_ by the arithmetic average of [S] during the considered time interval. This method was criticized by Karanth and Srivastava^33^, who concluded that the integrated equation yielded better results (in agreement with our conclusions and those of Cornish-Bowden^23^). Note, however, that these authors did not consider any possible experimental errors, but it remains surprising that none of these approaches has received the attention they deserved.

In our experimental approach, we used the P99 β-lactamase and the chromogenic substrate nitrocefin to continuously monitor the reaction time-courses. We did not use these time-courses, however, but we chose three values (i.e. 25, 50 and 60%) of conversion of the substrate at its lowest concentration (the so-called “constant time” strategy). The experimental results were consistent with the predictions based on the simulations: the (*K*_m_)_app_ and *V*_app_ values derived from the HMM equation increased with the percentage of substrate conversion but the latter significantly less than the former. By contrast, the *V* and *K*_m_ values obtained with the help of the integrated Eq. (4) were nicely homogeneous and in good agreement with the values obtained before^31^ under slightly different conditions.

Moreover, the experimental *V*_app_/*V* and (*K*_m_)_app_/*K*_m_ ratios were also very close to those predicted based on the simulations (Table 4). Our analysis shows that, if the four conditions mentioned in the introduction are fulfilled (i.e. the reaction is complete, the enzyme and the substrate are stable and there is no enzyme inactivation or inhibition by S or P), it is quite possible to obtain good estimates of the kinetic parameters with a single time point, if the data are sufficiently reliable (i.e. reproducible). It is important to evaluate the [S]_0_ values as accurately as possible and to obtain, for each [S]_0_ value, very precise measurements of [P] at the same *t* (although this is not absolutely necessary as long as *t* is also accurately determined). Indeed, the quality of the [P]/t measurements is essential and the utilisation of our approach cannot be an excuse for accepting inaccurate [P] estimations. Thus, if the assay is time-consuming (e.g. HPLC) our analysis suggests that it is better to perform, for each [S]_0_ value, four measurements at the same *t* rather than two measurements at two different *t* values. Moreover, it might be advantageous to utilise a larger proportion of substrate if 10% of the lowest [S]_0_ is near the detection limit of the assay method, hence leading to significant experimental errors.

A good test for the validity of the results is to compare the apparent values determined on the basis of the HMM equation (*V*_app_ and (*K*_m_)_app_) with the more realistic ones (*V* and *K*_m_) obtained with the help of the integrated Eq. (4). In all cases, the *V*_app_ and (*K*_m_)_app_ values (HMM equation) should be larger than those of *V* and *K*_m_ so that, as stated above, the *V*_app_/*V* and (*K*_m_)_app_/*K*_m_ ratios are good indicators of a kinetic model that fulfils the four conditions mentioned in the introduction. Moreover, both equations are applied to the very same experimental results. It is also interesting to note that, when reaction time courses are analysed on the basis of Eq. (4) (with [P] and *t* as variables), the results are very sensitive to minor systematic or experimental errors on [S]_0_, while the utilisation of the same equation as done here (with [P] and [S]_0_ as variables and a single time-point for each [S]_0_) results in much better estimations of *V* and *K*_m_.

Obviously, if Eq. (4) is expected to yield better results^34^, one might wonder if one should use the HMM Eq. (1) at all, but as stated above, the *V*_app_/*V* and (*K*_m_)_app_/*K*_m_ ratios are also good indicators of a kinetic model that fulfils the four conditions mentioned in the introduction, of the quality of the results and hence of the reliability of the derived *V* and *K*_m_ values. Of course, both equations are applied to the very same experimental results. Our approach does not eliminate the need for the usual necessary controls, for instance the possible detection of the non-enzymatic disappearance of S or of enzyme instability and the monitoring of at least one time-course to demonstrate the absence of hysteresis phenomena.

In conclusion, if one takes adequate precautions, it is perfectly possible to derive reliable *K*_m_ and *V* values based on single time-points obtained with a rather high degree of S conversion. This can be particularly useful when the assay method is very time-consuming, but also if the substrate is expensive or difficult to obtain. It is important to underline the fact that the HMM equation and Eq. (4) can be applied to the very same results, without the need to perform any additional experiments. This approach can also be particularly interesting to obtain preliminary data. Finally, it is also worth noting that this analysis is also valid when inhibition phenomena are studied, since the presence of the inhibitor influences the *V* and *K*_m_ values in ways characteristic of the type of inhibition. But this should be further studied.

In the past, with limited calculation methods available, linearisation of the rate equations was probably the only practical method available. With the presently available easy computational methods, utilisation of the integrated equations should be generalised (although, in the simple case, the HMM equation can yield very good results if one takes into account the overevaluations described in Table 1).

Note that the situation is significantly more complex in the case of multi-substrate reactions unless the variation of the concentration(s) of the other substrate(s) can be considered as negligible. This will be analysed in a further contribution. Similarly, reversible systems represent much more complex problems that are beyond the scope of the present analysis.

### Supplementary Information

Supplementary Information.

## Data Availability

The complete data can be found in 10.5281/zenodo.7528423.

## Abbreviations

HMM: Henri–Michaelis–Menten
HW: Hanes–Woolf
RE: Relative error
AE: Absolute error
SD: Standard deviation
SE: Standard error
P: Product
S: Substrate
