# Supplementary material for: The measurement of true initial rates is not always absolutely necessary to estimate enzyme kinetic parameters
Source: Sci Rep. 2023 Sep 12;13:15053. doi: 10.1038/s41598-023-41805-y (PMC10497622; doi:10.1038/s41598-023-41805-y)

**Enzyme kinetics: the initial rate dogma revisited.**

**Supplementary material.**

Jean-Marie Frère^1,2^*, Olivier Verlaine^2^ and André Matagne^1,2^*

^1^Enzymology and Protein Folding Laboratory, ^2^Centre for Protein Engineering, InBioS, University of Liège, Building B6C, Quartier Agora, Allée du 6 Août, 13, 4000 Liège (Sart-Tilman), Belgium.

^*^Authors to whom correspondence should be addressed.

Tel.: +32 (0)4 3663419

E-mails: amatagne@uliege.be, jmfrere@uliege.be

The complete data can be found in: doi.org/10.5281/zenodo.7528423

# Simulations and introduction of errors.

[P]/t values were calculated with equation 2.

Relative errors (RE) were then introduced up to 2, 5 or 10 % according to the following algorithm:

$$min=1-\left( \frac{RE}{100} \right);max=1+\left( \frac{RE}{100} \right)$$

$$t_{error}=\left( \left( max-min \right)\times Rnd+min \right)\times t$$

where Rnd is a value in the interval [0,1].

In each case, 18 runs were performed.

Absolute errors (AE) were introduced up to 20 % of the [P]/t values obtained at the lowest [S]_0_ value.

$$perror= Absolue error=20 a=\frac{-V_{min}\times perror}{100}; b=-a$$

$$error=a+\left[ \left( b-a \right)\times rand \right]; rand=random number between 0 et 1$$

$$V_{error}=V+error$$

where Rnd is a value in the interval [0,1].

In each case, 18 runs were performed.

# Experimental procedures.

## Compounds and enzymes

Nitrocefin was purchased from BD Diagnostics (Erembodegem, Belgium). The β-lactamase from *Enterobacter cloacae* P99 was prepared as described^29,31^.

## Kinetic studies

All experiments were performed in 10 mM HEPES buffer, pH 8.2, and the reaction time-courses were monitored at 30°C and 485 nm, using a Specord 200 spectrophotometer (Analytik Jena) equipped with a thermostatically controlled cell holder (500 µl cells, 1-cm optical pathway), as described ^31^. The enzyme, at a final concentration of 0.6 nM, was added to the substrate solution in a total volume of 450 µL, containing 0.2 M NaCl and 50 µg·ml^‑1^ of bovine serum albumin.

## Data analysis

The Prism 9 software (GraphPad) was used for least-squares analysis of the linearized data. In all other cases, the Matlab software was used. Errors were calculated throughout as standard deviations (SDs) on average values and standard errors (SEs) on the parameters obtained by fitting the data to the relevant equations.

## Results of the simulations.

Exact [P]/t values were calculated with the help of the integrated equation 2.

Table S1 shows examples for 2 [S]_0_ concentrations, 0.35 and 2.45. Table S2 shows the results obtained with the “constant percentage of conversion” strategy.

Table S3 compares 5 sets of 18 simulations with errors up to 10 % and 70 % substrate conversion (constant time strategy).

Figure S1 shows the [P]/t vs [S]_0_ plot at 50 % conversion of the substrate (“constant percentage of conversion” strategy).

Figure S2 shows how the curvature detected in Figure 1 at 70 % substrate conversion (main text) can disappear in the presence of experimental errors.

## Experimental results.

Table S4 summarizes the results obtained with nitrocefin concentrations ranging from 11 to 64 µM.

# Legends to the Tables

**Table S1.** Simulations of *t* vs [P]. A and B: [S]_0_ = 0.35 and (A) 10 % or (B) 50 % final transformation of the substrate. C and D: [S]_0_ = 2.45 and (C) 50 % final conversion of the substrate or (D) with a final time equal to that necessary to reach 50 % transformation at [S]_0_ = 0.35. To simplify the table, the *t* values (and [P] in part D) were limited to 4 decimal places. All units are arbitrary (see text).

**Table S2.** Values of *V_app_* and (*K*_m_*)_app_* deduced from [P]/*t vs* [S]_0_ plots for increasing percentages of substrate conversion. The % of substrate conversion was the same at all S concentrations. Analysis according to the Hanes-Woolf linearization yielded exactly the same results. All units are arbitrary (see text).

**Table S3.** Comparison of 5 sets of 18 simulations. 70 % substrate conversion, 10 % maximum error (“constant time” strategy).

**Table S4**. [P]/*t* values (*t* = 48.9 s) for the hydrolysis of nitrocefin by the P99 β-lactamase. A total of 18 experiments were performed and the averaged values are displayed here.

# Legends to the Figures.

**Figure S1.** [P]/t vs [S]_0_ plot at 50 % conversion of the substrate at all [S]_0_ values (“constant percentage of conversion” strategy). The curve was drawn according to equation 1 with the *V_app_* and (*K*_m_*)_app_* values found in Table S2.

**Figure S2.** Example showing how the upward curvature in the Hanes-Woolf plot can become impossible to detect in the presence of experimental errors. Substrate conversion is 70 % in all cases.

**A.** No error. HW plots: *V_app_* = 1.27 ± 0.08, (*K*_m_*)_app_* = 2.21 ± 0.26. Integrated equation: *V* = 1.00 ± 0.002, *K_m_* = 1.00 ± 0.008

**B.** Errors up to 5 %. HW plot: *V_app_* = 1.23 ± 0.20, (*K*_m_*)_app_* = 2.1 ± 0.59. Integrated equation: *V* = 0.96 ± 0.11, *K_m_* = 0.91 ± 0.19.

**C.** Errors up to 10 %. HW plot: *V_app_* = 1.35 ± 0.31, (*K*_m_*)_app_* = 2.37 ± 0.9. Integrated equation: *V* = 0.97 ± 0.19, *K_m_* = 0.91 ± 0.34.

**Table S1**

| **[S]_0_ = 0.35** | | | | **[S]_0_ = 2.45** | | | |
| --- | --- | --- | --- | --- | --- | --- | --- |
| **A**  **10 % conversion of S** | | **B**  **50 % conversion of S** | | **C**  **50 % conversion of S** | | **D**  **final *t* = 0.8681** | |
| ***t*** | **[P]** | ***t*** | **[P]** | ***t*** | **[P]** | ***t*** | **[P]** |
| 0 | 0 | 0 | 0 | 0 | 0 | 0 | 0 |
| 0.0194 | 0.005 | 0.0991 | 0.025 | 0.2491 | 0.175 | 0.1196 | 0.0845 |
| 0.0390 | 0.010 | 0.2041 | 0.050 | 0.5042 | 0.350 | 0.2403 | 0.1691 |
| 0.0588 | 0.015 | 0.3162 | 0.075 | 0.7661 | 0.525 | 0.3629 | 0.2536 |
| 0.0788 | 0.020 | 0.4365 | 0.100 | 1.0365 | 0.700 | 0.4866 | 0.3381 |
| 0.0991 | 0.025 | 0.5668 | 0.125 | 1.3168 | 0.875 | 0.6120 | 0.4227 |
| 0.1196 | 0.030 | 0.7096 | 0.150 | 1.6097 | 1.050 | 0.7391 | 0.5072 |
| 0.1404 | 0.035 | 0.8681 | 0.175 | 1.9181 | 1.225 | 0.8681 | 0.5917 |

**Table S2.**

| **% Substrate conversion** | ***V*_app_** | **(*K*_m_)_app_** | ***V_app_/(K_m_)_app_*** |
| --- | --- | --- | --- |
| 10 | 1.001 ± 0.003 | 1.06 ± 0.007 | 0.94 ± 0.005 |
| 20 | 0.999 ± 0.001 | 1.11 ± 0.004 | 0.90 ± 0.004 |
| 30 | 0.999 ± 0.003 | 1.18 ± 0.008 | 0.85 ± 0.005 |
| 40 | 1.000 ± 0.001 | 1.28 ± 0.004 | 0.78 ± 0.003 |
| 50 | 0.999 ± 0.003 | 1.38 ± 0.008 | 0.73 ± 0.005 |

**Table S3.** Comparison of 5 sets of 18 simulations.

|  | **Hanes-Woolf** | | | | **Integrated HMM equation** | | | |
| --- | --- | --- | --- | --- | --- | --- | --- | --- |
|  | ***K_m_* (range)** | ***K_m_* (average)** | ***V* (range)** | ***V* (average)** | ***K_m_* (range)** | ***K_m_* (average)** | ***V* (range)** | ***V* (average)** |
| Set 1 | 1.44 – 2.73 | 2.11 ± 0.41 | 0.99 – 1.60 | 1.24 ± 0.14 | 0.70 – 1.23 | 0.91 ± 0.12 | 0.84 – 1.13 | 0.96 ± 0.07 |
| Set 2 | 1.56 – 3.33 | 2.29 ± 0.46 | 1.01 – 1.68 | 1.29 ± 0.16 | 0.76 – 1.15 | 0.96 ± 0.13 | 0.85 – 1.09 | 0.97 ± 0.07 |
| Set 3 | 1.78 – 2.89 | 2.29 ± 0.46 | 1.14 – 1.49 | 1.31 ± 0.16 | 0.84 – 1.16 | 0.98 ± 0.13 | 0.93 – 1.09 | 1.00 ± 0.08 |
| Set 4 | 1.60 – 3.11 | 2.14 ± 0.46 | 1.04 – 1.61 | 1.23 ± 0.15 | 0.73 – 1.12 | 0.90 ± 0.13 | 0.85 – 1.10 | 0.94 ± 0.08 |
| Set 5 | 1.66 – 3.46 | 2.19 ± 0.49 | 1.05 – 1.65 | 1.26 ± 0.17 | 0.77 – 1.27 | 0.96 ± 0.14 | 0.86 – 1.14 | 0.98 ± 0.08 |
| No error |  | 2.21 ± 0.27 |  | 1.27 ± 0.09 |  | 1.002 ± 0.004 |  | 1.001 ± 0.002 |

**Table S4.**

| **[S]_0_ (µM)** | **[P]/t (µM/min)** | **%Substrate converted** |
| --- | --- | --- |
| 11.1 | 6.87 ± 0.33 | 51.2 |
| 22.1 | 12.0 ± 0.33 | 44.2 |
| 35.0 | 15.8 ± 0.2 | 36.8 |
| 52.2 | 18.9 ± 0.8 | 29.5 |
| 64 | 21.7 ± 1.0 | 27.6 |

**Figure S1**

**Figure S2**


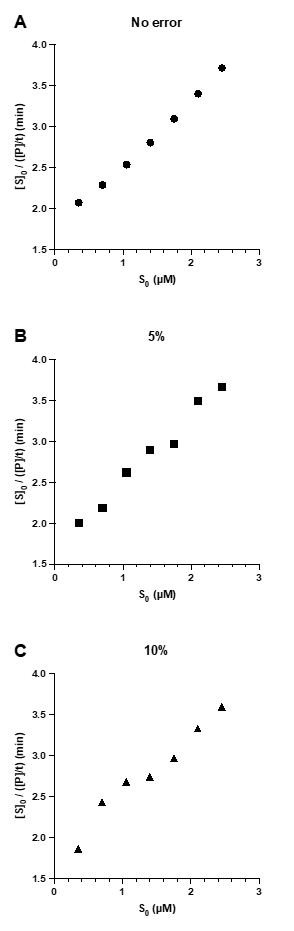

Supplement: Supplementary file 1 — Supplementary Information. [file 41598_2023_41805_MOESM1_ESM.docx]
